# Effects of *Bacillus megaterium* on growth performance, serum biochemical parameters, antioxidant capacity, and immune function in suckling calves

**DOI:** 10.1515/biol-2020-0106

**Published:** 2020-12-31

**Authors:** Jun Yao, Lili Wang, Wenju Zhang, Mengjian Liu, Junli Niu

**Affiliations:** College of Animal Science & Technology, Shihezi University, Shihezi 832003, China; School of Bioengineering, Dalian University of Technology, Dalian 116000, China

**Keywords:** suckling calve, *Bacillus megaterium*, biochemical parameter, antioxidant, immune function

## Abstract

**Background:**

This study was conducted to investigate the effects of *Bacillus megaterium* on growth performance, serum biochemical parameters, antioxidant capacity, and immune function in suckling calves.

**Methods:**

In total, 20 1-day-old Holstein calves with similar body weight (BW) and good health condition were randomly assigned into two groups with ten replicates per group and one calf per replicate. The control group (CON group) was fed a basal diet, whereas the *B. megaterium* group (BM group) was fed the basal diet supplemented with 500 mg/day/head of *B. megaterium* (10^10^ CFU/g) for 28 days.

**Results:**

The results revealed that the BM group showed an increase in final BW, daily weight gain, and feed-to-gain ratio (*p* < 0.05) and a decrease in diarrhea rate. Moreover, the concentrations of serum cholesterol and high-density lipoprotein decreased (*p* < 0.05) in the BM group compared with the CON group at 28 days. The level of serum glutathione was higher (*p* < 0.05) in the BM group than that of the CON group at 14 days, whereas the level of serum malondialdehyde decreased (*p* < 0.01) in the BM group compared with the CON group at 28 days. In addition, compared with the CON group (*p* < 0.05), the concentrations of serum IgA, IgM, IgG, and IL-4 were higher, whereas the concentration of serum TNF-α decreased in the BM group at 28 days.

**Conclusion:**

*B. megaterium* had beneficial effects on the improvement of growth performance, immune function, and intestinal oxidative status of suckling calves.

## 1 Introduction

The feeding management of suckling calves is essential to the cattle industry. After the calves are born, the living environment changes for them greatly, and the resistance of calves to the external environment might be low. If the calves are not immune enough to resist external bacteria or lack of nutrition, it will lead to gastrointestinal dysfunction, diarrhea, and even death [1,2]. Although the resistance of calves increases after 3 weeks of growth, they are still highly susceptible to infectious diseases. Therefore, to improve the production performance and health level of calves, we must pay attention to the regulation of immunity and reduce the risk of disease. At present, antibiotics are often used in production as a means to enhance the disease resistance of animals. However, the long-term use of antibiotics has brought many serious problems such as drug resistance and drug residues [3,4].

Because of the problems faced by the transitional use of antibiotics, people are trying to find new safe and effective green additives [5,6,7,8]. *Bacillus megaterium* is a probiotic with rich enzyme production and strong resistance to pathogenic bacteria. It belongs to the genus *Bacillus*. At present, its research on animal husbandry is still in the preliminary stage. The research results of some Chinese scholars in livestock, poultry, and aquatic animals show that the addition of *B. megaterium* in diet can improve the feed intake, daily gain, feed conversion rate, and growth performance [9,10,11,12]. However, studies on the application and mechanism of *B. megaterium* in young ruminants, such as calves and lambs, have been rarely reported. This research aimed to evaluate the effects of *B. megaterium* on growth performance, serum biochemical parameters, antioxidant capacity, and immune function in sucking calves and then attempted to preliminarily explore the possible mechanisms of action. We hope to provide data support for the application of *B. megaterium* in calf feeding.

## 2 Materials and methods

### 2.1 Test strain

*B. megaterium* was obtained from the R&D center of COFCO (Changji) Grain and Oil Industry Co., Ltd, Changji, China (ccj-bac-meg1801) and preserved in our laboratory. Our previous studies have shown that the strain had excellent resistance to artificial gastric juice and intestinal juice, inhibition to *Escherichia coli*, and ability to produce enzymes [13].

### 2.2 Animals and experimental design

In total, 20 1-day-old Holstein calves with similar body weight (BW) and good health condition were randomly assigned into two groups with ten replicates per group and one calf per replicate. The control group (CON group) was fed a basal diet, whereas the *B. megaterium* group (BM group) was fed the basal diet supplemented with 500 mg/day/head of *B. megaterium* (10^10^ CFU/g) for 28 days. The basal diet included milk, starter diet, and alfalfa. The composition of the starter diet is shown in Table 1, and the nutritional level of each diet is shown in Table 2. Calves were fed 4 L of colostrum within 1 h after birth, 6 L of cow milk at 40°C for 0–7 days, and 8 L of cow milk thrice (08:30, 14:30, and 20:30) a day for 8–28 days. *B. megaterium* was mixed in milk and fed. The calves began to feed the starter diet and ate alfalfa freely at the age of 7 days.

**Table 1:** Composition of the starter diet (air-dry basis)

Ingredients	
Item	Content (%)
Soybean meal	25
Extruded soybean	13
Dried whey	5
Corn	25
Extruded corn	17.9
Wheat bran	10
CaHPO_4_	0.8
Salt	0.5
Limestone	1.8
Premix	1
Total	100

Premix provides the following per kilogram of the starter diet: V A 15,000 IU, V D 65,000 IU, V E 50 IU, Fe 90 mg, Mn 60 mg, Cu 12.5 mg, Zn 100, I 2.0 mg, Co 0.5 mg, and Se 0.3 mg.

**Table 2:** Nutrient levels of milk, starter diet, and alfalfa (air-dry basis)

Item	Milk	Starter diet	Alfalfa
DM (%)	12.63	91.27	94.63
CP (%)	3.19	18.02	14.67
EE (%)	3.92	4.19	1.53
Crude ash (%)	0.69	7.57	9.35
Ca (%)	0.12	1.09	1.47
P (%)	0.09	0.58	0.31
GE (MJ/kg)	2.72	17.65	17.98

Nutrient levels were all measured values.


**Ethical approval:** The research related to animal use has been complied with all the relevant national regulations and institutional policies for the care and use of animals and has been approved by the animal ethical committee of the First Affiliated Hospital of Medical College of Shihezi University (ethical number: A2019-152-01).

### 2.3 Determination of growth performance

The BWs of calves were measured per replicate basis at 1, 14, and 28 days. The daily feeding amount and remaining amount of milk, starter diet, and alfalfa were recorded during the whole trial. The average daily gain (ADG), average daily dry matter intake (DMI), and feed-to-gain ratio (F/G) were then calculated. Instances of the diarrhea in each calf were recorded daily to calculate the rate of diarrhea according to the fecal scoring standard in Table 3 [14]. If the score is ≥3, it will be regarded as one diarrhea.

**Table 3:** Fecal score standards

Degrees	Appearance	Score
Normal	Thick in consistency	1
Normal	Thick in consistency, but less thick	2
Abnormal	Thin but not watery	3
Abnormal	Watery	4
Abnormal	Watery with abnormal coloring	5

\text{Diarrhea rate (} \% )=\hspace{3em}\left(\frac{\text{whole number of calves with diarrhea × diarrhea days}}{\text{whole number of calves × experimental days}}\right)\hspace{.5em}\times \hspace{.5em}100.

At 14 and 28 days of the trial, 20 mL of blood was collected from the jugular vein before morning feeding. The collected blood samples were centrifuged at 3,000 rpm for 15 min at 4°C to separate the serum, which was frozen at −20°C until further analysis.

### 2.4 Determination of serum biochemical parameters

Serum total protein (TP), albumin (ALB), globulin (GLB), urine nitrogen (UN), glucose (GLU), cholesterol (CHOL), triglycerides (TG), high-density lipoprotein (HDL), and low-density lipoprotein (LDL) concentrations and alkaline phosphatase (ALP), alanine aminotransferase (ALT), aspartate aminotransferase (AST), and lactate dehydrogenase (LDH) activities were measured by the automatic biochemical analyzer (Fully, diagnostic systems, Italy) according to the instructions of the corresponding kits (Nanjing Jiancheng Bioengineering Institute, Nanjing, China).

### 2.5 Determination of antioxidative capacity

Serum catalase (CAT), total antioxidant capacity (T-AOC), and total superoxide dismutase (T-SOD) activities and glutathione (GSH) and malondialdehyde (MDA) levels in serum were measured according to the previously described methods [15] using commercial analysis kits (Nanjing Jiancheng Bioengineering Institute, Nanjing, China).

### 2.6 Determination of serum immune parameters

The levels of IgA, IgG, IgM, IL-1β, IL-2, IL-4, IL-6, IL-10, IFN-γ, and TNF-α in serum were detected strictly according to the manufacturer’s instructions using the commercially available ELISA kits (Nanjing Jiancheng Bioengineering Institute, Nanjing, China; No. E027-1-1, E026-1-1, E025-1-1, H001, H003, H005, H007, H009, H025, and H052, respectively).

### 2.7 Statistical analysis

Experimental data were analyzed by one-way ANOVA using SPSS version 21.0 software (SPSS Inc., Chicago, IL, USA). The differences between the two groups were compared by Tukey’s test. All data were expressed as mean ± SD. When *p* < 0.05, there were significant differences between the two groups. The diarrhea rate of calves was only compared in percentage.

## 3 Results

### 3.1 Growth performance

The effect of *B. megaterium* on the growth performance of sucking calves is shown in Table 4. During the whole trial, *B. megaterium* supplementation improved BW, ADG, and F/G, especially for 1–14 days (*p* < 0.05). There was no significant difference in DMI between the two groups (*p* > 0.05). The diarrhea rate of the BM group was 4.59% lower than that of the CON group.

**Table 4:** Effect of *B. megaterium* on growth performance of calves

Item	CON	BM
**BW (kg)**		
1 day	38.33 ± 5.37	37.95 ± 6.18
14 days	41.38 ± 7.10^b^	43.02 ± 7.99^a^
28 days	47.80 ± 8.62^b^	49.94 ± 10.01^a^
**ADG (g/day)**		
1–14 days	218.01 ± 92.05^b^	362.33 ± 80.65^a^
15–28 days	458.36 ± 83.87	494.40 ± 50.37
1–28 days	338.19 ± 30.22^b^	428.37 ± 52.19^a^
**DMI (g/day)**		
1–14 days	830.62 ± 30.28	801.06 ± 80.81
15–28 days	1098.72 ± 69.90	1082.26 ± 73.52
1–28 days	964.67 ± 22.53	941.66 ± 19.93
**F/G (g/g)**		
1–14 days	3.81 ± 0.28^a^	2.21 ± 0.19^b^
15–28 days	2.40 ± 0.32	2.19 ± 0.06
1–28 days	2.85 ± 0.36^a^	2.20 ± 0.14^b^
**Diarrhea rate (%)**		
1–28 days	15.45	10.86

There was no significant difference in the same row of data without superscripts (*p* > 0.05). Different small letters showed significant difference (*p* < 0.05).

### 3.2 Serum biochemical parameters

The effect of *B. megaterium* on serum biochemical parameters of sucking calves is shown in Table 5. There were no significant differences in serum biochemical parameters between the two groups at 14 days (*p* > 0.05). The concentrations of serum CHOL and HDL decreased (*p* < 0.05) in the BM group compared with the CON group at 28 days.

**Table 5:** Effect of *B. megaterium* on serum biochemical parameters of calves

Item	14 days		28 days	
	CON	BM	CON	BM
ALB (g/L)	30.09 ± 3.01	29.40 ± 3.23	31.13 ± 2.94	33.08 ± 1.90
GLB (g/L)	28.08 ± 1.48	27.93 ± 1.55	27.82 ± 1.87	27.65 ± 2.61
ALB/GLB (g/g)	1.07 ± 0.02	1.05 ± 0.02	1.12 ± 0.03	1.20 ± 0.04
TP (g/L)	58.17 ± 3.90	57.33 ± 4.27	58.95 ± 3.85	60.73 ± 3.13
ALT (U/L)	13.07 ± 0.32	14.22 ± 0.25	15.06 ± 0.76	16.13 ± 0.65
AST (U/L)	46.68 ± 4.03	45.43 ± 3.16	50.29 ± 4.48	49.34 ± 4.86
ALT/AST (U/U)	3.57 ± 0.19	3.19 ± 0.11	3.40 ± 0.17	3.06 ± 0.17
ALP (U/L)	46.99 ± 3.12	57.67 ± 2.33	43.40 ± 8.92	39.00 ± 5.99
TG (mmol/L)	0.36 ± 0.01	0.33 ± 0.01	0.38 ± 0.02	0.37 ± 0.01
CHOL (mmol/L)	1.54 ± 0.15	1.32 ± 0.12	1.78^a^ ± 0.16	1.39^b^ ± 0.13
HDL (mmol/L)	1.95 ± 0.08	1.79 ± 0.08	2.18^a^ ± 0.06	1.87^b^ ± 0.05
LDL (mmol/L)	0.35 ± 0.02	0.32 ± 0.01	0.29 ± 0.01	0.26 ± 0.01
LDH (U/L)	499.50 ± 21.53	497.67 ± 19.30	568.40 ± 25.14	539.33 ± 20.30
UN (mmol/L)	2.67 ± 0.16	2.80 ± 0.21	2.69 ± 0.46	2.11 ± 0.26
GLU (mmol/L)	5.49 ± 0.12	5.75 ± 0.17	4.92 ± 0.26	5.06 ± 0.19

There was no significant difference in the same row of data without superscripts (*p* > 0.05). Different small letters showed significant difference (*p* < 0.05).

### 3.3 Antioxidative capacity

**Figure 1:**
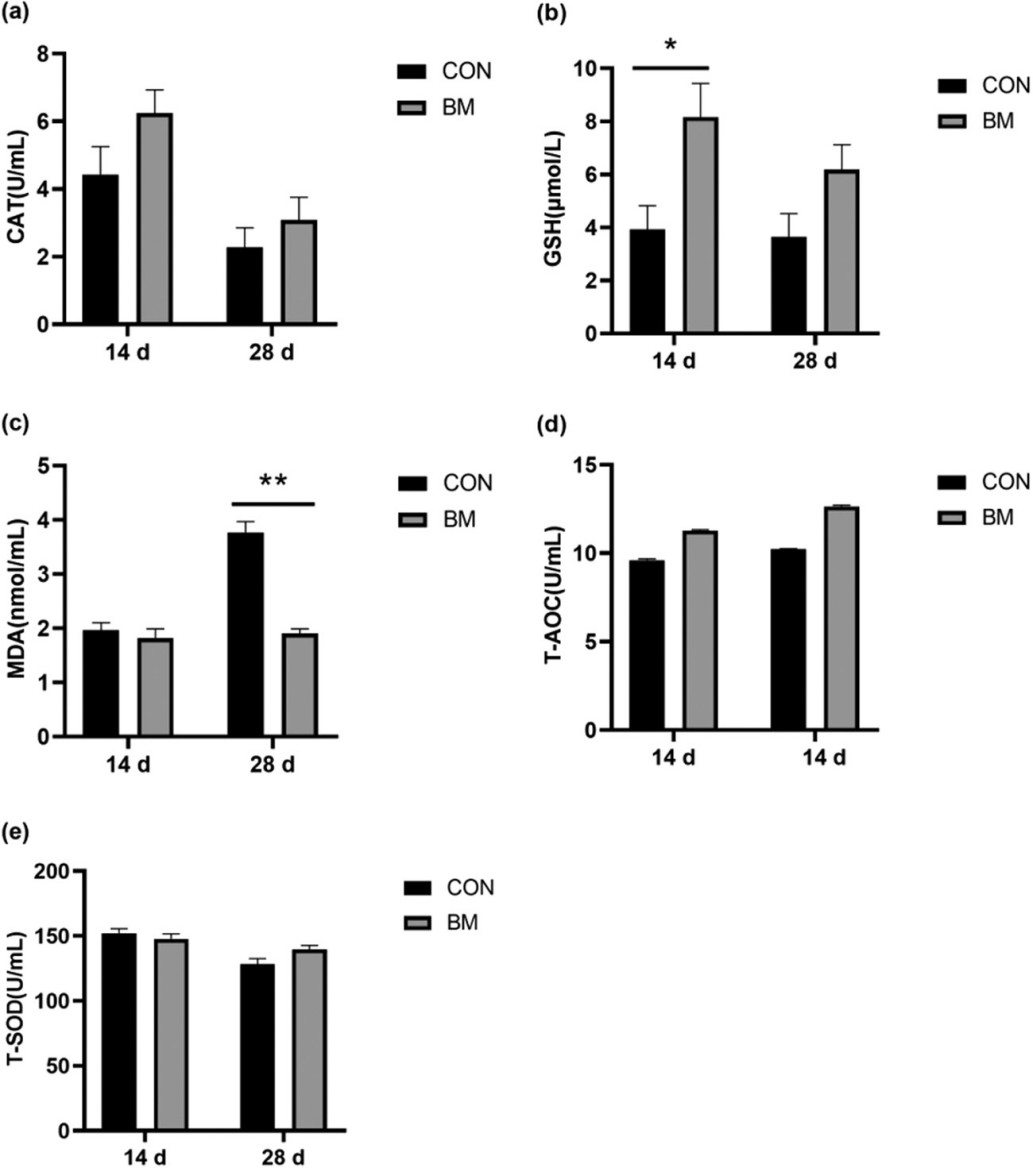
Effect of *B. megaterium* on serum antioxidative capacity of calves. (a) CAT activity. (b) The level of serum GSH. (c) The level of serum MDA. (d) T-AOC level. (e) T-SOD activity. *Significant difference (*p* < 0.05). **Extremely significant difference (*p* < 0.01).

The effect of *B. megaterium* on antioxidative capacity of sucking calves is shown in Figure 1. No significant differences in serum CAT activity, T-SOD activity, and T-AOC level were observed (Figure 1a, d, and e). The level of serum GSH was higher (*p* < 0.05) in the BM group than that of the CON group at 14 days, whereas the level of serum MDA significantly decreased (*p* < 0.01) in the BM group compared with the CON group at 28 days (Figure 1b and c).

**Figure 2:**
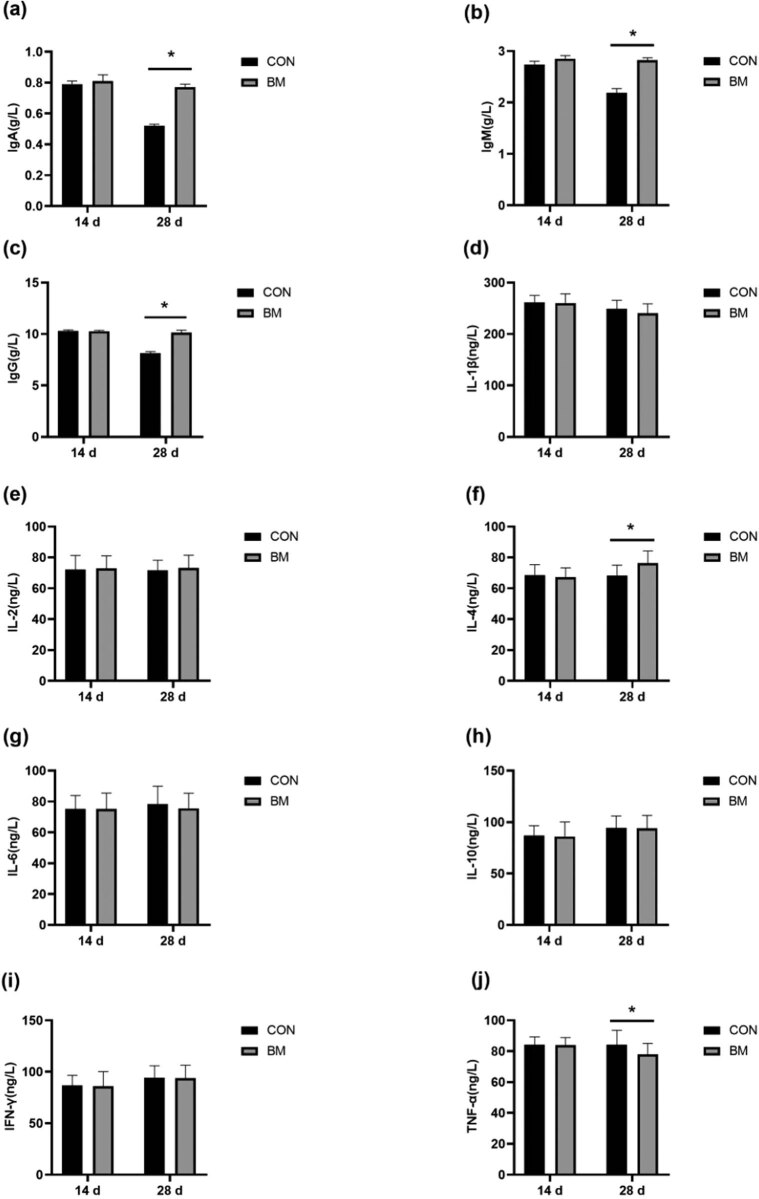
Effect of *B. megaterium* on serum immune parameters of calves. The concentrations of serum IgA (a), IgM (b), IgG (c), IL-1β (d), IL-2 (e), IL-4 (f), IL-6 (g), IL-10 (h), IFN-γ (i) and TNF-α (j). *Significant difference (*p* < 0.05).

### 3.4 Serum immune parameters

The effect of *B. megaterium* on serum immune parameters of sucking calves is shown in Figure 2. There were no significant changes in the immune parameters between the two groups (*p* > 0.05). The concentrations of serum IgA, IgM, IgG, and IL-4 were higher (*p* < 0.05) than that of the CON group (Figure 2a, b, c, and f), whereas the concentration of serum TNF-α decreased (*p* < 0.05) compared with the CON group at 28 days (Figure 2j).

## 4 Discussion

The growth performance of calves is an important index to evaluate farmers’ feed additives. Probiotics, as feed additives, can regulate intestinal flora, enhance gastrointestinal digestion and absorption, and then improve the growth performance and feed conversion rate of animals [16,17,18]. A previous study showed that adding 0–12 g/day *Bacillus natto* fermentation in lactating dairy cow diets had no significant effect on milk protein and milk fat rates, but could significantly improve the milk yield and feed conversion rate [19]. Similar findings obtained by adding *Bacillus licheniformis* to dairy diets could improve feed conversion and milk protein rate [20]. Another previous study also showed that adding 10^9^ CFU/kg probiotics could significantly increase the ADG of weaned piglets and reduce the F/G and the diarrhea rate [21]. Using 0.2% probiotics in the diet could significantly improve ADG and F/G of growing and grown pigs and promote the quality of pork [22]. In the present research, we found that the ADG and F/G improved and the diarrhea rate decreased compared with the CON group, which indicated that *B. megaterium* promoted the growth and alleviated the stress of newborn calves, consistent with previous reports. These effects of promoting growth and anti-stress were more prominent in the early period (1–14 days) of sucking calves. Possible reasons for the growth and development of suckling calves promoted by *B. megaterium* are: on one hand, the addition of *B. megaterium* in the diet can effectively degrade the anti-nutritional factors in the diet, eliminate their anti-nutritional effects, and promote the digestion and decomposition of nutrients by the body so as to improve the feed conversion rate; on the other hand, when *B. megaterium* enters and is planted in the gastrointestinal tract of calves, it can produce extracellular enzymes with strong activity, such as protease and amylase, and produce enzymes that degrade non-starch polysaccharides in plant feed. These enzymes can degrade the corresponding nutrients in the feed and improve the digestibility of nutrients in feed. Therefore, the digestion and absorption rate are increased. At the same time, *B. megaterium* can quickly consume oxygen in the intestine, promote the growth of *Lactobacillus* and *Bifidobacterium*, and the short-chain fatty acids produced can effectively reduce the intestinal pH and limit the reproduction of harmful bacteria. Therefore, the diarrhea rate of calves is reduced.

Serum biochemical parameters can be used as the key index to predict the health of calves. Probiotics can decrease the incidence of diarrhea, lactose intolerance, and serum cholesterol [23,24]. Previous studies found that feeding rats with *Lactobacillus* and *Bifidobacterium* could promote the metabolism of triglyceride, total cholesterol, and total bile acid [25]. *Lactobacillus acidophilus* had significant antitumor activity and cholesterol-lowering effect [26]. In this trial, there was no significant difference in most serum biochemical parameters between the BM group and CON group. At 28 days, the concentration of CHOL and HDL in the serum of the BM group was significantly lower than that of the CON group, which was similar to the previous studies. Meanwhile, the concentration of LDL had a decreasing trend. The CHOL in blood is mainly carried and transported by LDL. HDL helps to clear the cholesterol in cells, and their concentrations are related. Therefore, the changing trends of CHOL, LDL, and HDL concentrations are the same [27]. The change of CHOL concentration in blood is an important index reflecting whether the metabolism of lipids is normal or not, and it can also reveal the stress of the body. The results showed that *B. megaterium* could alleviate the lipid transport barrier caused by the great change of living environment or the transformation from liquid diet to solid diet, and ensure the normal metabolism of lipid in calves, which had no negative impact on the health of calves.

The antioxidant capacity of the animal defense system can also reflect the animal’s health. Under normal circumstances, the production of free radicals in animals and the scavenging ability of the antioxidant defense system to free radicals maintain a good dynamic balance. When animals are sick or suffering from stress, excessive free radicals and oxides in the body will cause oxidative damage to the body [28]. T-SOD is a substance that can scavenge free radicals and peroxides and reduce hydroxyl radicals. GSH is the most important peptide antioxidant, which can clear away free radicals, detoxify, and maintain the integrity of erythrocyte membrane and cellular immunity. As an important antioxidant and free radical scavenger in the body, GSH can improve the immunity and disease resistance of the body. The level of GSH is a vital index to measure the antioxidant capacity of the body [29]. T-AOC is a representative parameter to judge the antioxidant function of the body [30]. MDA, as the end product of lipid oxidation, can show the degree of lipid peroxidation mediated by oxygen-free radicals. In this research, we found that the addition of *B. megaterium* in diet could significantly increase the level of serum GSH of sucking calves at 14 days and significantly reduce the level of MDA of sucking calves at 28 days. It may be because of the accumulation of GSH in the BM group in the first 14 days. At 28 days, the generation of MDA was inhibited by the antioxidant effect of a large number of GSH, which made the level of MDA in the BM group significantly lower than that of the CON group. There were no significant changes in other antioxidant indicators in the serum. However, Gong et al. [31] found that three strains of *Bacillus* considerably increased serum T-SOD activity, T-AOC level, and SOD and CAT activities in the liver of broilers. There were many possible reasons that could lead to inconsistent test results. Probiotic strains, probiotic doses, dietary composition, environment, and animal species could all influence the test outcomes. Moreover, the feeding time might have been too short resulting in *B. megaterium* not being able to fully exert its antioxidant function. However, all parameters of the BM group were numerically greater than the CON group. Therefore, it was proved that *B. megaterium* could reduce the decrease in antioxidant function caused by the stress of calves. The reason may be that there are many anti-nutritional factors in the protein raw materials which can destroy the immune organs of the body and reduce its defense function. However, *B. megaterium* can promote the body to secrete antioxidant enzymes through its own secretion or as an activator, resulting in the improved anti-oxidative ability of the body.

Immunoglobulins can bind antigens, activate complement, regulate humoral immune function, reflect the immune level of the body, and even directly reflect the changes of immune organ function [32]. IgA, IgG, and IgM are important immunoglobulins in calves. Probiotics can lead to an increase of the animal immunoglobulin levels and enhanced body immunity [33,34,35]. In the present research, we observed that the concentrations of IgA and IgM in the serum of suckling calves were significantly increased by adding *B. megaterium* to milk at the age of 28 days. Dabiri et al. [36] found that the number of lymphocytes and immunoglobulins of lambs in the probiotic group increased during the 12th week of the experiment, similar to the findings of this study. The immune ability of lambs in the experimental group was significantly higher than that of the CON group. This suggested that *B. megaterium* could change the concentration of immunoglobulin in animal serum by regulating cellular immunity so as to enhance the immune function of the body, and alleviate the decrease in disease resistance caused by the great change of living environment of newborn calves or the transformation from liquid diet to solid diet. *B. megaterium* may secrete immunoglobulin to improve the immune function of calves. Immunoglobulin has antibacterial and antiviral functions, which may be a mechanism of *B. megaterium* to improve the immune function of animals.

Cytokines in serum play an important role in inflammatory response [37], which is the expression of cellular immune function. Cytokines have a certain influence on the differentiation of immune cells and inflammation of the body. IL-1β, IL-2, IL-6, IF-γ, and TNF-α are pro-inflammatory factors. IL-4 and IL-10 are anti-inflammatory factors [38]. IL-4 can reduce the production of IFN-γ, whereas IL-10 can restrain the release of TNF-α and IL-6 from macrophages and dendritic cells [39]. Probiotics can regulate the gene and protein expression of cytokines or enhance the toxicity of cytokines to play an immune-regulatory role [40,41]. Previous studies found that *Lactobacillus* could significantly downregulate IL-1β gene expression in the ileum of piglets 14 days after weaning and reduce IL-6, IL-10, and TNF-α mRNA expression [42]. *Lactobacillus plantarum* could alleviate the increase of chemokine IL-8 induced by TNF-α in Caco-2 cells [43]. *Lactobacillus sakei* could promote the expression of pro-inflammatory factors IL-1β and TNF-α in Caco-2 cells [44]. *Lactobacillus rhamnosus* GG could abnormally reduce elevated IL-1β and IL-6 in piglet serum caused by *E. coli* and improve humoral immunity of piglets [45]. In this trial, the results were similar to previous studies showing that TNF-α in the serum of the BM group was significantly lower than that of the CON group, whereas IL-4 in the serum was significantly higher than that of the CON group at 28 days. It may be because of the inflammatory response of the test calves during the sucking period. The immune function of the calf organism was improved with the coordination of *B. megaterium* over time. The anti-inflammatory effect of *B. megaterium* may be through the activation of toll-like receptor 2 (TLR-2) in intestinal epithelial cells of animals so as to regulate the secretion of pro-inflammatory factors and anti-inflammatory factors by the NF-κB signaling pathway, and then reduce the infection of pathogenic bacteria [46,47,48]. More in-depth experiments are required to verify whether the above assumptions are correct.

In conclusion, adding *B. megaterium* to the diet could promote the growth performance of sucking calves by altering lipid metabolism decreasing CHOL and LDL levels, enhancing anti-oxidative and anti-stress capacity, and stimulating the immune function of the body. Therefore, *B. megaterium* can be used as a new probiotic preparation in the feeding of suckling calves.
